# Rates and trends of childhood acute lymphoblastic leukaemia: an epidemiology study

**DOI:** 10.1038/s41598-020-63528-0

**Published:** 2020-04-21

**Authors:** Ameer Kakaje, Mohammad Marwan Alhalabi, Ayham Ghareeb, Bahjat Karam, Bassam Mansour, Bayan Zahra, Othman Hamdan

**Affiliations:** 1 Faculty of medicine, Damascus University, Damascus, Syria; 2 Haematology department, Children’s University Hospital, Damascus University, Damascus, Syria

**Keywords:** Cancer, Risk factors, Signs and symptoms

## Abstract

Acute lymphoblastic leukaemia (ALL) is the most common childhood cancer and has a high survival rate when properly managed. Prognosis is correlated with many factors such as age, gender, white blood cell (WBC) count, CD10, French-American-British (FAB) classification, and many others. Many of these factors are included in this study as they play a major role in establishing the best treatment protocol. This study aims to demonstrate clinical and laboratory features of childhood ALL in Syria. They were treated at Children’s University Hospital, the only working major cancer centre in Syria at the time of the study. Data of 203 patients who aged 0–14 years were obtained for this study. Most patients (48.8%) aged (5–9) years with a male predominance (60.9%). The major features for ALL included lymphadenopathy (82.9%), presenting with systemic symptoms (74.9%), T-ALL subclass (20.2%), L2 FAB classification (36.1%), low educational levels for fathers (53%) and mothers (56.2%), having a high risk (48.4%), and having a duration of symptoms before evaluation for more than 4 weeks (42.6%). Only three (1.5%) patients had normal full blood counts (FBC) and only one (0.5%) patient had an isolated high WBC count at time of presentation. Most patients had either abnormal platelet count (89.3%) or low haemoglobin level (88.8%) when presenting with only (2.0%) having normal levels for both. This suggests that having normal haemoglobin and platelet count can be used for quick screening in crisis time like in Syria for prioritising patients. Many prognostic factors were significantly different from medical literature which emphasises the importance of local studies in the developping countries. This study included a high prevalence of T-all, L2 FAB classification, high-risk and other variables which require further studies to evaluate the aetiology of these features, especially that treatment protocols may have a higher mortality in developing countries when not adjusted to local variables.

## Introduction

Acute lymphoblastic leukaemia (ALL) is the most common cancer in childhood, with a prevalence up to 25% of cancers in children who are under the age of 15 years^1^. Although ALL is curable, many parts of the world may not have access to modern treatment. Approximately eight to nine of every ten children that have ALL are considered long-term survivors and cured in developed countries, but these reults markedly differ in developping countries^2,3^. These positive outcomes are due to having access to top treatments at the most advanced institutions^2^. Although the five-year survival rate is 93.5% when using the newest protocols and top chemotherapy, some cases of relapse still occur^4^. Nevertheless, top treatment cannot be accessed by all countries as many factors may get involved such as resource scarcity^5^. Few studies were concerned with paediatric ALL in the Middle East^6^. Little is known about childhood ALL in the Middle East and further studies are needed to establish standard data for future regional collaborative research as it provides a baseline for future protocols in ALL as they needed to be adjusted to the local variables. This raises a challenge as it is not easy to conduct medical research such as medical trials in developing countries due to the unavailability of porper funding and institutions which makes this quite challenging. In this article, the epidemiology and characterstics of ALL patients along with ALL variables such as prognostic risk and subtype are studied. Our study aims to define the risk factors and features associated with ALL in Syria.

## Materials and methods

### Study design

Our cross-sectional study was conducted in the Children’s University Hospital of Damascus University. The data was collected from patients’ records and covered the period between 21^st^ August 2017 and 21^st^ August 2018. This hospital is the major paediatric cancer centre among two centres in Syria and provides free healthcare to patients. The other centre was in Aleppo and was not working properly in the study period due to the conflict in Aleppo, resulting in most of leukaemia cases to be referred to Damascus.

### Sampling and data collecting

This study included children with ALL who aged 0 to 14 years. ALL was diagnosed before initiating chemotherapy by bone marrow aspiration and immune phenotyping. Information was obtained from the hospital’s records which were taken by the physicians at time of diagnosis and information was provided by the child’s caregiver.

### Demographics and family history

Data about general characteristics of patients such as age, gender, and province of origin was recorded (Table 1). Caregivers were asked by hospital physicians to determine the history of cancer and leukaemia in the family. Family history was obtained based on the family of the affected child having malignancies in their direct family.

**Table 1:** Characteristics of Children with ALL in Syria.

Characteristic	Count	Percentage (CI 95%)	Characteristic	Count	Percentage (CI 95%)
**Gender**			**ALL subtype**		
Male	123	60.9 (54–67.8)	B ALL	158	79.8 (73.8–85.4)
Female	79	39.1 (32.2–46)	T ALL	40	20.2 (14.6–26.2)
**Age (years)**			**WBC when diagnosed (cells/L)**		
0–4	74	36.6 (29.7–43.1)	1.5×10^9^ and less	5	2.5
5–9	98	48.5 (42.1–55.9)	(1.5–11.5) × 10^9^	91	45.7
10–14	30	14.9 (9.9–20.3)	11.5 × 10^9^ and above	103	51.8
**Place of living**			**Hemoglobin levels when diagnosed (g/dl)**		
Damascus	15	7.7	11–16	24	12
Rif-Dimashq	36	18.5	11–7	106	53
Aleppo	11	5.6	7 and less	70	35
Homs	20	10.3			
Hama	22	11.3			
Deir ez-Zur	17	8.7			
Ar Raqqah	11	5.6			
Al Hasakah	24	12.3	**Platelets count (cells/L)**		
Daraa	10	5.1			
As Suwayda	8	4.1	More than 400 × 10^9^	4	2
Quneitra	1	0.5	(150 to 400) × 10^9^	21	10.6
Latakia	4	2.1	(150 to 50) × 10^9^	63	31.8
Tartus	4	2.1	(50 to 20) × 10^9^	60	30.3
Idlib	12	6.2	Less than 20 × 10^9^	50	25.3
Mother education level*			**CXR**		
Low	91	56.2	Mediastinal enlargement or lymphadenopathies	32	18.4
Medium	55	34	Negative	142	81.6
High	16	9.9			
Father education level*			**CD 10**		
Low	88	53	81% and more	104	58.1
Medium	51	30.7	21–80%	36	20.1
High	27	16.3	20% and less	39	21.8
**Main presenting symptom:**			**FAB classification**		
Systemic symptoms	140	74.9	L1	93	58.9 (51.3–65.8)
Lymphadenopathy	20	10.7	L2	57	36.1 (28.5–44.3)
Hepato-splenomegaly	5	2.7	L3	8	5.1 (1.9–8.9)
Bruising	16	8.6			
Accidental	6	3.2			
**Hepato-splenomegaly:**			**Prognostic risk**		
Positive	139	73.2	Standard	96	51.6 (44.6–58.6)
Negative	51	26.8	High	90	48.4 (41.4–55.4)
**Lymphadenopathy**			**Duration of symptoms before evaluation (weeks)**		
Positive	165	82.9	0–2		
Negative	34	17.1	2–4	51	25.9
			4+	62	31.5
				84	42.6
**Family history**					
Positive	20	10.6 (6.3–15.9)			
Negative	169	89.4 (84.1–93.7)			

CI: Confidence interval.

### French-American-British (FAB) classification

A skilled professor in haematology was involved in determining FAB classification^7^ for each ALL patient whether it was L1, L2 or L3. As FAB does not have independent prognostic significance and it is subjective, it is no longer recommended^1^. FAB classification was the first classification for ALL^8^, and it is based on morphology and cytochemical staining. However, it remains effective despite cytogenetic tests as it can add diagnostic accuracy in some cases^9^ Furthermore, using FAB system is convenient in developing countries as it is easy to conduct in regular labs and does not require much resources^10^. It is also used when there is no alternative which is why it is used in Syria.

### Risk determination

Berlin-Frankfurt-Münster (BFM) risk groups determination was used^1^. However, in this study standard and intermediate risk groups were merged into one group that has both characteristics and treated as intermediate risk. This change is more convenient due to lack of resources to determine the genes and it is easier for management and application. In addition, long-term treatment response was beyond this study scope.

Patient’s prognostic risk was defined as standard or high. Poorer prognosis is correlated with age of younger than 1 year and older than 10 years, white blood cell (WBC) count higher than 50 × 10^9^ cells/L at time of diagnosis, extramedullary disease, biologic and cytogenetic changes such as having Philadelphia chromosome, T-ALL, positive cerebrospinal fluid (CSF) and testicular involvement, inability to tolerate standard chemotherapy, slow-rate response to initial therapy, the speed and how low leukemia cell count drops after initial therapy, minimal residual disease (MRD) and bone marrow aspiration and FAB determination in the beginning of treatment; these were all considered in the determination of each patient’s prognosis to conduct the correct chemotherapy protocol^8^. MRD was determined by a blood smear on day 8 and steroid response.

Patients with hereditary risk factors such as Down syndrome, neurofibromatosis, Bloom syndrome, Fanconi anemia, ataxia telangiectasia, Li-Fraumeni syndrome, and constitutional mismatch repair deficiency were excluded from this study.

### Definitions

Systemic symptoms were defined as having fever, anorexia, weight loss, or fatigue. Chest x-ray (CXR) was considered positive when it had a mediastinal enlargement or hilar lymphadenopathy. WBC count of (1.5–11.5 × 10^9^ cells/L), haemoglobin level of (11–16 g/dL), and platelet count of (150–400 × 10^9^ cells/L) were considered normal. A positive family history is when a direct family member has a history of malignancy, regardless of type of cancer or age of presentation.

We defined having a positive CD10 as having 21% or higher CD10 on flow cytometry. Educational levels were divided into 3 groups as in Syria these three groups tend to significantly differ; low education level is for a parent whose higher degree is elementary or lower, medium educational level is when the highest degree is the ninth or 12^th^ grade, and finally high educational level is when having a university degree or higher.

### Genetic testing

No routine genetic testing was conducted due to unavailability of resources and other countries boycot the high-tech materials and medications which made them very expensive for the government to obtain for this centre. However, genetic testing was, conducted when a hereditary syndrome was highly suspected and therefore these patients could be excluded from this study, but genes prevalence such as *Philadelphia* gene is not valid to use in this study as genetic testing is not routinely done, and therefore the data was not retrieved.

### Consent and ethical approval

Informed written consent was taken before using and publishing the data. It was taken from the parent and/or the legal guardian of the child. The study was approved by the ethics committee of Damascus University. We confirm that all research was performed in accordance with relevant guidelines/regulations.

### Data analysis

Data was processed by the software IBM SPSS version 26 for Windows (SPSS Inc, IL, USA). The statistical analysis used was Chi-square test for determination of statistical significant differences within the groups. We measured odds ratios (ORs) and the 95% confidence intervals when comparing groups by using Mantel–Haenszel test by using the same software. When two-tailed P value was less than 0.05, the results were considered to be statistically significant.

## Results

### Characteristics of the sample

**Figure 1:**
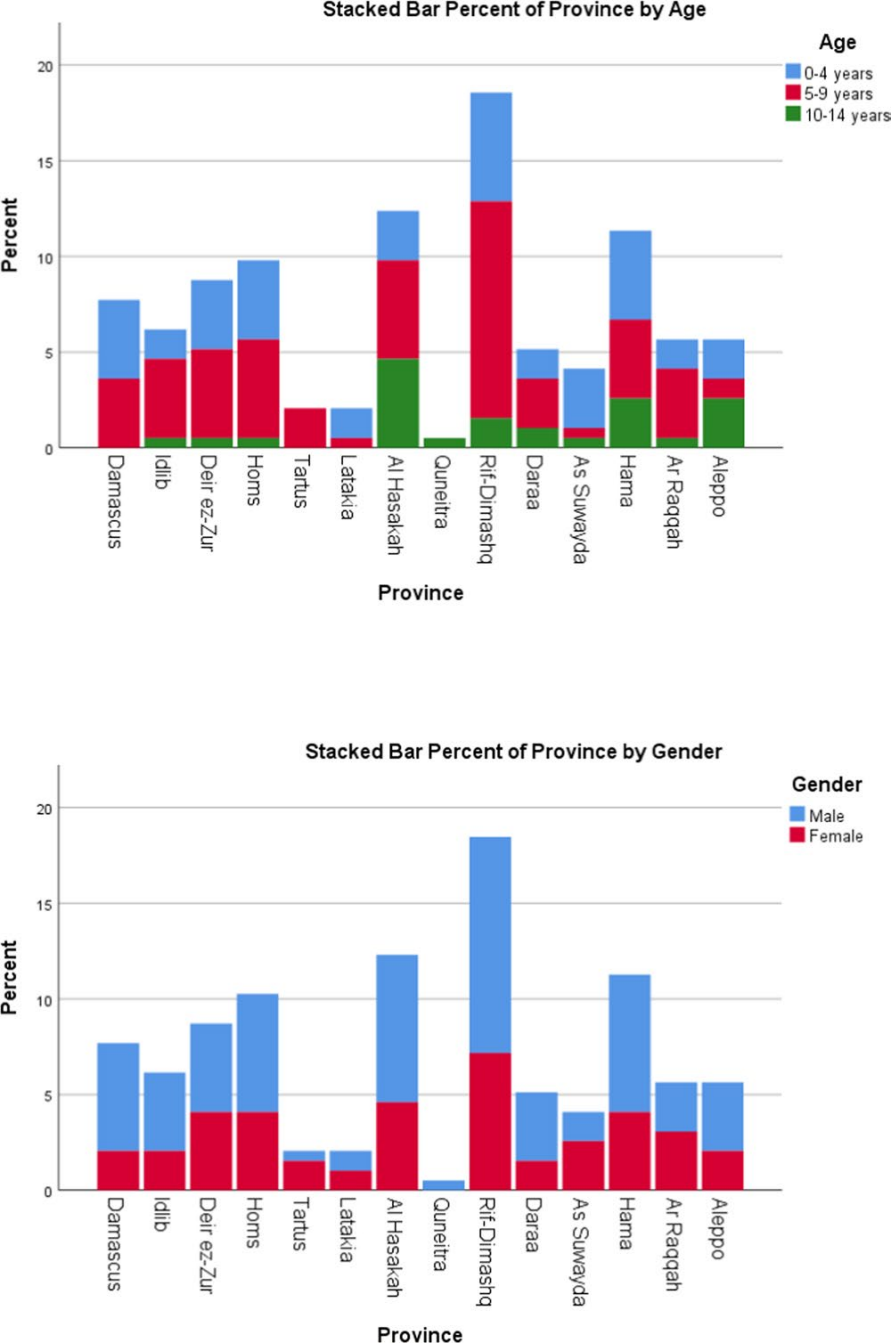
Showing age, gender, and province of origin for ALL patients.

Our study was conducted on 203 ALL patients who aged (0–14) years. The peak age in our study was (5–9) years comprising 48.5% of the cases with a male predominance (60.9%). Characteristics of ALL children in Syria, including gender, age, geographic distribution, parents’ educational level, main presenting symptom, hepato-splenomegaly, lymphadenopathy, ALL-subtype, haemoglobin, WBC and platelet count when diagnosed, CXR, CD10, FAB classification and prognostic risk are demonstrated in (Table 1). Patients distribution in Syrian provinces is shown in (Fig. 1) according to the province of origin.

Date of the full blood count (FBC) of 197 patients was recorded; only three (1.5%) patients had normal FBC (normal haemoglobin, WBC count and platelet counts) when diagnosed and only one (0.5%) patient had an isolated high WBC count with normal haemoglobin and platelet counts. However, only 21 (10.7%) patients had normal platelet counts, only 22 (11.2%) patients had normal haemoglobin, and only four (2.0%) of the patients having normal haemoglobin and platelet counts when diagnosed.

Having systemic symptoms (74.9%), anaemia (88%) with 35% having severe anaemia, low platelet count (87.4%) with 25.3% having platelet count lower than (20 × 10^9^ cells/L), lymphadenopathy (82.9%), and hepato-splenomegaly (73.9%) were the most frequent observations in our study.

### Variables according to gender

**Figure 2:**
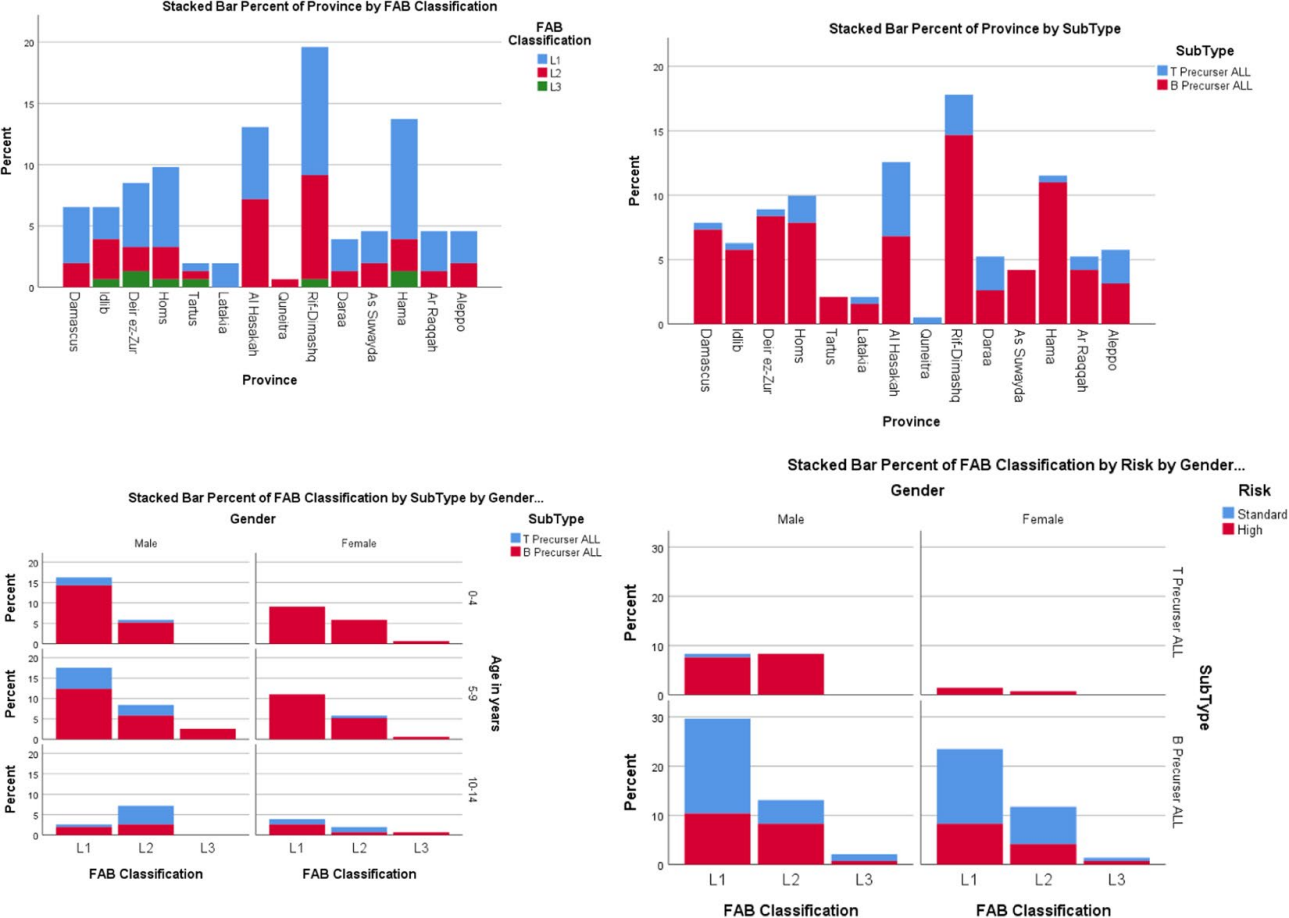
Showing gender, province of origin, subtype, FAB classification of ALL patients, and risk.

Comparison between males and females with ALL characteristics is demonstrated in Table 2. T-ALL was more frequently correlated with male gender P = 0.0019 (OR, 3.750; 95% CI 1.565–8.986). Lymphadenopathies were less common in females comparing to males P = 0.0286 (OR, 0.439; 95% CI 0.208–0.928) and males had a longer duration of symptoms before evaluation (more than 4 weeks) when compared to females P = 0.0145 (OR, 2.054; 95% CI 1.149–3.671). No statistically significant difference was found when comparing gender with having family history, parents’ educational levels, hepato-splenomegaly, haemoglobin, WBC and platelet count, CXR, CD10 positivity, prognostic risk and FAB classification (P > 0.05). Classification and ALL subtype according to province of origin are demonstrated in Fig. 2.

**Table 2:** Comparing males and females with ALL in children in Syria.

Characteristic	Male	Percentage (CI 95%)	Female	Percentage (CI 95%)	*P* value
**Family history**					
Negative	104	90.4 (84.3–95.7)	65	87.8	NS
Positive	11	9.6 (4.3–15.7)	9	12.2	
**Mother Education level**					
Low	61	59.2	30	50.8	NS
Medium	32	31.1	23	39	NS
High	10	9.7	6	10.2	
**Father Education level**					
Low	56	54.4	32	50.8	NS
Medium	29	28.2	22	34.9	NS
High	18	17.5	9	14.3	
**Subtype**					
B ALL	88	72.7 (63.6–81.0)	70	90.9 (83.1–96.1)	0.0019
T ALL	33	27.3 (19.0–36.4)	7	9.1 (3.9–16.9)	
**Hepato-splenomegaly**					
Negative	27	23.3	24	32.4	NS
Positive	89	76.7	50	67.6	
**Lymphadenopathy**					
Negative	15	12.4	19	24.4	0.0286
Positive	106	87.6	59	75.6	
**Duration of symptoms before evaluation (weeks)**					
0–2	35	29.7	16	20.3	NS
2–4	41	34.7	21	26.6	0.0518^a^
4+	42	35.6	42	53.2	
**WBC when diagnosed (cells/L)**					
1.5×10^9^ and less	1	0.8	4	5.1	NS
(1.5–11.5) × 10^9^	53	43.8	38	48.7	NS
11.5 × 10^9^ and above	67	55.4	36	46.2	
**Hemoglobin levels when diagnosed (g/dl)**					
11–16	17	14.0)	7	8.9	NS
11–7	63	52.1	43	54.4	NS
7 and less	41	33.9	29	36.7	
**Platelets count (cells/L)**					
More than 400 000 × 10^9^	3	2.5	1	1.3	NS
(150 000 to 400 000) × 10^9^	11	9.2	10	12.7	NS
Less than 150 000 × 10^9^	105	88.2	68	86.1	
**CXR**					
Mediastinal enlargement or lymphadenopathies	81	21.4	10	14.1	NS
Normal		78.6	61	85.9	
**CD 10**					
21% and more	86	76.8	54	80.6	NS
Negative	26	23.2	13	19.4	
**Prognostic risk**					
Standard	53	46.5 (37.7–56.1)	43	59.7 (48.6–70.8)	0.0786
High	61	53.5 (43.9–62.3)	29	40.3 (29.2–51.4)	
**FAB classification**					
L1	56	58.3 (49.0–68.7)	37	59.7 (46.8–71.0)	NS^b^
L2	35	36.5 (27.1–45.8)	22	35.5 (24.2–46.8)	
L3	5	5.2 (1.0–9.4)	3	4.8 (0.0–11.3)	

CI: Confidence interval.

^a^when comparing duration of symptoms before evaluation of 4 weeks and more with less than 4 weeks, P = 0.0145.

^b^P value was only calculated between L1 and L2.

### Variables according to age groups

Comparison between age groups with characteristics of ALL is demonstrated in Table 3. T-ALL was found more frequently than B-ALL in the oldest age group (10–14) when compared with the age group (5–9) P = 0.0030 (OR, 3.690; 95% CI 1.529–8.929) or with the age group (0–9) P = 0.0001 (OR, 4.975; 95% CI 2.160–11.494). The prognostic risk was found to be higher in older patients (10–14) than the younger patients (5–9) P = 0.0001 (OR, 7.500; 95% CI 2.405–23.386) and the youngest patients (0–9) P < 0.0001 (OR, 8.492; 95% CI 2.812–25.643). CD10 was found to be negative more frequently in the age group (0–9) when compared with (10–14) age group P = 0.0146 (OR, 0.352; 95% CI 0.149, 0.833).

**Table 3:** Comparison of Characteristics of ALL children comparing with age groups.

	4 years and below	Percentage (CI 95%)	5–9 years	Percentage (CI 95%)	*P* value^a^	10–14 years	Percentage (CI 95%)	*P* value^b^	0–9 years	*P value*^*c*^
**Gender**										
Male	45	60.8 (48.6–71.6)	59	60.2 (51.0–68.4)	NS	19	63.3 (46.7–80.0)	NS	104	NS
Female	29	39.2 (28.4–51.4)	39	39.8 (31.6–49.0)		11	36.7 (20.0–53.3)		68	
**Subtype**										
B-cell ALL	66	89.2 (81.1–95.9)	76	80.9 (72.3–89.4)	NS	16	53.3 (36.7–70.0)	0.003	142	0.0001
T-cell ALL	8	10.8 (4.1–18.9)	18	19.1 (10.6–27.7)		14	46.7 (30.0–63.3)		26	
**Risk category**										
Standard	42	61.8 (50.0–72.1)	50	55.6 (45.6–65.6)	NS	4	14.3 (3.6–28.6)	0.0001	92	<0.0001
High	26	38.2 (27.9–50.0)	40	44.4 (34.4–54.4)		24	85.7 (71.4–96.4)		66	
**Duration of symptoms before evaluation (weeks)**										
0–2	20	27.4	23	24.5	*NS*	8	26.7	*NS*	43	*NS*
2–4	25	34.2	29	30.9	*NS*	8	26.7	*NS*	54	*NS*
4+	28	38.4	42	44.7		14	46.7		70	
					*NS*	0	0	*NS*	5	*NS*
**WBC (cells/L)**										
1.5×10^9^ and less	1	1.4	4	4.1	*NS*	14	48.3	*NS*	77	*NS*
(1.5–11.5) × 10^9^	31	42.5	46	47.4	NS	15	51.7	NS	88	NS
11.5 × 10^9^ and above	41	56.2	47	48.5	*NS*	7	23.3	*NS*	17	*NS*^*d*^
**Haemoglobin (g/dl)**									89	
11+	6	8.2	11	11.3	NS	17	56.7	NS	64	NS^d^
11–7	36	49.3	53	54.6	NS	6	20	NS		NS
Under 7	31	42.5	33	34		2	7.1		2	
**Platelets count (cells/L)**										
400** + **× 10^9^	0	0	2	2.1	NS	4	14.3	NS	17	NS
(150–400) × 10^9^	10	13.7	7	7.2	NS	22	78.6	NS	151	NS
150 × 10^9^ and less	63	86.3	88	90.7						
**CXR**										
Normal	53	84.1	70	81.4	NS	19	76	NS	123	NS
Abnormal	10	15.9	16	18.6		6	24		26	
**Mother education level**										
Low	25	41.7	53	67.9	*0.0018*	13	54.2	*NS*	78	*NS*
Medium	29	48.3	19	24.4	*NS*	7	29.2	*NS*	48	*NS*
High	6	10	6	7.7		4	16.7		12	
**Father education level**					*0.0766*			*NS*	76	*NS*
Low	28	47.5	48	58.5	*NS*	12	48	*NS*	45	*NS*
Medium	24	40.7	21	25.6		6	24		20	
High	7	11.9	13	15.9		7	28			
**CD 10**									28	
Negative	10	15.2	18	21.2	NS	11	39.3	0.0571	123	0.0146
21% and above	56	84.8	67	78.8		17	60.7			
**FAB classification**										
L1	39	66.1 (54.2–78.0)	44	59.5 (47.3–70.3)	NS^e^	10	40.0 (20.0–60.0)	0.0488^e^	83	0.0252^e^
L2	19	32.2 (20.3–44.1)	24	32.4 (21.6–43.2)		14	56.0 (36.0–76.0)		43	
L3	1	1.7 (0.0–5.1)	6	8.1 (2.7–14.9)		1	4.0 (0.0–12.0)		7	
**Hepato-splenomegaly**										
Negative	16	24.6	21	22.1	NS	14	46.7	0.009	37	0.0076
Positive	49	75.4	74	77.9		16	53.3		123	
**Lymphadenopathy**						*7*	23.3		27	
Negative	14	19.7	13	13.3	NS	23	76.7	NS	142	NS
Positive	57	80.3	85	86.7						
**Family history**										
Negative	62	92.5 (85.1–98.5)	84	89.4 (83.0–94.7)	NS	23	82.1 (64.4–92.9)	NS	146	NS
Positive	5	7.5 (1.5–14.9)	10	10.6 (5.3–17.0)		5	17.9 (7.1–35.6)		15	

ALL: acute lymphoblastic leukaemia; NS: not significant; FAB: French–American–British classification.

Different total count for subjects is due to missing data.

^a ^P value is between (0–4) and (5–9) age groups.

^b ^P value is between (5–9) and (10–14) age groups.

^c ^P value is between (0–9) and (10–14) age groups.

^d ^When comparing normal haemoglobin with abnormal haemoglobin between age groups of (0–9) and (10–14) years, P = 0.0383.

^e ^P value is calculated between L1 and L2 FAB classification.

L2 was found more frequently than L1 in (10–14) age group when compared with (5–9) age group P = 0.0488 (OR, 2.567; 95% CI 0.991–6.649) or with (0–9) age group P = 0.0252 (OR, 2.702; 95% CI 1.108–6.588). Less hepato-splenomegaly was found in the older age group (10–14) when compared with the younger age group (5–9) P = 0.0090 (OR, 0.324; 95% CI 0.136–0.771) or with the age group (0–9) P = 0.0076 (OR, 0.344; 95% CI 0.154–0.770). However, no statistically significant difference was found when comparing gender, duration of symptoms before evaluation, haemoglobin, WBC and platelet count, CXR, parents’ educational level, lymphadenopathy, or family history with any age group (P > 0.05).

### Variables according to ALL subtype

Comparison between T-ALL and B-ALL with characteristics of ALL is demonstrated in Table 4. WBC count was found to be less frequently higher than normal (higher than 11 × 10^9^ cells/L) in B-ALL P < 0.0001 (OR, 0.200; 95% CI 0.083–0.482). However, haemoglobin was found to be more frequently low in B-ALL patients P = 0.0012 (OR, 4.421; 95% CI 1.723–11.345). CXR was found to be less positive in B-ALL P < 0.0001 (OR, 0.109; 95% CI 0.046–0.259). CD10 was found to be more frequently positive in B-ALL patients P < 0.0001 (OR, 32.500; 95% CI 12.462–84.755) and prognostic risk to be lower in B-ALL patients P < 0.0001 (OR, 0.016; 95% CI 0.002–0.122). L1 was found less frequently in T-ALL patients P = 0.0401 (OR, 0.432; 95% CI 0.192–0.974). No statistically significant difference was found between T-ALL and B-ALL when compared with family history, parents’ educational level, hepato-splenomegaly, time until diagnosis, and platelet count P > 0.05.

**Table 4:** Comparing Characteristics of T-ALL and B-ALL in children in Syria.

Characteristic	T-ALL	Percentage (CI 95%)	B- ALL	Percentage (CI 95%)	*P* value
**Family history**					
Negative	33	84.6 (74.4–94.9)	133	90.5 (85.7–95.2)	NS
Positive	6	15.4 (5.1–25.6)	14	9.5 (4.8–14.3)	
**Mother Education level**					
Low	23	63.9	68	55.7	NS
Medium	10	27.8	41	33.6	NS
High	3	8.3	13	10.7	
**Father Education level**					
Low	19	54.3	68	53.5	NS
Medium	10	28.6	39	30.7	NS
High	6	17.1	20	15.7	
**Hepato-splenomegaly**					
Negative	9	23.7	39	26.4	NS
Positive	29	76.3	109	73.6	
**Lymphadenopathy**					
Negative	3	7.9	31	19.7	0.0841
Positive	35	92.1	126	80.3	
**Duration of symptoms before evaluation (weeks)**					
0–2	16	43.2	34	21.8	0.0972
2–4	11	29.7	49	31.4	NS^a^
4+	10	27	73	46.8	
**WBC (cells/L)**					
1.5×10^9^ and less	1	2.6	3	1.9	NS
(1.5–11.5) × 10^9^	7	18.4	83	52.9	0.0001^b^
11.5 × 10^9^ and above	30	78.9	71	45.2	
**Haemoglobin levels when diagnosed (g/dl)**					
11–16	12	30.8	12	7.6	0.0012^c^
11–7	19	48.7	84	53.5	NS
7 and less	8	20.5	61	38.9	
**Platelets count (cells/L)**					
More than 400 × 10^9^	2	5.4	2	1.3	NS
(150 to 400) × 10^9^	5	13.5	16	10.2	NS
150 × 10^9^ and less	30	81.1	139	88.5	
**CXR**					
Mediastinal enlargement or lymphadenopathies	18	51.4	14	10.4	<0.0001
Normal	17	48.6	121	89.6	
**CD 10**					
21% and more	9	25	130	91.5	<0.0001
Negative	27	75	12	8.5	
**Prognostic risk**					
Standard	1	2.8 (0.0–8.3)	95	63.8 (55.7–71.1)	<0.0001
High	35	97.2 (91.7–100.0)	54	36.2 (28.9–44.3)	
**FAB classification**					
L1	14	46.7 (26.8–66.7)	79	63.2 (55.2–72.0)	0.0401^d^
L2	16	53.3 (33.3–73.2)	39	31.2 (23.2–39.2)	
L3	0	0	7	5.6 (1.6–9.6)	

^a^ P = 0.0290 when calculated between duration of symptoms before evaluation for more than 4 weeks and less than 4 weeks.

^b^ when comparing normal WBC count with abnormal WBC count, T-ALL had abnormal WBC count more frequently than B-ALL P = 0.0001.

^c^ when comparing normal haemoglobin and low haemoglobin, B-ALL had low haemoglobin more frequently P < 0.0001.

^d^ P value is calculated between L1 and L2 FAB classification.

### Variables according to risk group

Comparison between high risk and low risk patients with characteristics of ALL is demonstrated in Table 5. Positive family history was found more frequently in patients with standard risk P = 0.0438 (OR, 2.916; 95% CI 0.992–8.570). However, CD10 was found less frequently in patients with high risk P < 0.0001 (OR 0.129; 95% CI 0.050–0.331) and L2 was found more frequently in patients with high risk P = 0. 0227 (OR 2.267; 95% CI 1.114–4.613). No statistically significant difference when comparing patient risk with their parents’ educational level (P > 0.05).

**Table 5:** Characteristics of High and standard risk categories patient and L1 and L2 in ALL in Syrian children.

Characteristic	High risk	Percentage%	Standard risk	Percentage%	*P* value
**Family history**					
Negative	74	85.10%	83	94.30%	0.0438
Positive	13	14.90%	5	5.70%	
**Mother Education level**					
Low	43	55.80%	42	59.10%	NS
Medium	28	36.40%	20	28.20%	NS
High	6	7.80%	9	12.70%	
**Father Education level**					
Low	42	53.20%	40	54.80%	NS
Medium	26	32.90%	19	26.00%	NS
High	11	13.90%	14	19.20%	
**CD 10**					
21% and more	51	63.70%	82	93.20%	<0.0001
Negative	29	36.30%	6	6.80%	
**FAB classification**					
L1	40	55.60%	51	73.90%	0.0227
L2	32	44.40%	18	26.10%	
**Characteristic**	**L1**	**Percentage%**	**L2**	**Percentage%**	**P value**
**WBC (cells/L)**					
1.5×10^9^ and less	2	2.10%	1	1.80%	NS
(1.5–11.5) × 10^9^	37	39.80%	21	38.20%	NS
11.5 × 10^9^ and above	54	58.10%	33	60.00%	
**Hemoglobin levels when diagnosed (g/dl)**					
11–16	10	10.80%	9	16.10%	NS
11–7	43	46.20%	31	55.30%	NS
7 and less	40	43.00%	16	28.60%	
**Platelets count (cells/L)**					
More than 400 × 10^9^	1	1.10%	2	3.70%	NS
(150 to 400) × 10^9^	8	8.60%	5	9.30%	NS
150 × 10^9^ and less	84	90.30%	47	87.00%	
**Mother Education level**					
Low	40	52.60%	26	56.50%	NS
Medium	30	39.50%	15	32.60%	NS
High	6	7.90%	5	10.90%	
**Father Education level**					
Low	39	51.30%	27	56.30%	NS
Medium	28	36.80%	10	20.80%	0.0307
High	9	11.90%	11	22.90%	
**CD 10**					
21% and more	70	84.30%	33	66.00%	0.0143
Negative	13	15.70%	17	34.00%	
**Family history**					
Negative	76	87.40%	49	90.70%	NS
Positive	11	12.60%	5	9.30%	

### Variables according to FAB classification

Comparison between L1 and L2 patients with characteristics of ALL is demonstrated in Table 5. CD10 was found less frequently positive in L2 patients P = 0.0143 (OR 0.361; 95% CI 0.157–0.829) and L2 patients had a higher probability of having a father with high educational level P = 0.0307 (OR 3.422; 95% CI 1.096–10.690). However, no statistically significant difference was found when comparing L1 and L2 with haemoglobin, WBC and platelet count, mother educational level and family history.

### Other variables

Having hepato-splenomegaly was more frequently correlated with high WBC count P = 0.0055 or with abnormal WBC count P = 0.0057, and with higher rate of low platelets P = 0.0095 or abnormal platelet P = 0.0079 compared with normal platelets. Lymphadenopathy was found to be correlated with high WBC count P = 0.0123 or abnormal WBC count P = 0.0144, and with higher prognostic risk P = 0.0418. In patients with low platelets (less than 150 × 10^9^ cells/L), having hepato-splenomegaly was found to be more frequently correlated with even lower platelet count (less than 20 × 10^9^ cells/L) P = 0.0452.

## Discussion

### Age and gender

The mean age group of our study is slightly older than the (3–6) years reported by the Multi-Institutional International Collaborative Study (CALLME1) by the Middle East Childhood Cancer Alliance (MECCA) in which they comprised 33.8%^6^, older than what was found in the US of (1–4) years comprising (42.9%)^11,12^, and one international study that covered 184 countries which found the peak age to be (0–4) years^13^, but was close to the age reported in the Tehran study with a mean age of 5.5 years^14^ and a Brazilian study which had the average age of diagnosis of 6.3 ± 0.5 years^15^. Gender ratio of M/F in our study was (1.56:1) which was slightly higher than the CALLME1 study (1.4:1), the US study (1.35:1), the international study (1.4:1), and the Tehran study (1.32:1), but lower than what was found in the Brazilian study (1.9:1) but there was no significant difference (P > 0.05) when comparing our study with all previous studies.

### Symptoms, FBC and organomegaly

Most patients presented with systemic symptoms (74.9%) similar to the Tehran study in which the patients had fever (51.2%), organomegaly (31.4%) and pallor (19.2%) and to the Brazilian study which found that hepatomegaly, splenomegaly, fever and lymphadenopathy were the most common clinical features. Most patients (42.6%) in our study required more than 4 weeks to get diagnosed which was similar to the CALLME1 study which found that the mean time before evaluation to be 1.35 months. However, in our study (25.9%) of patients needed less than 2 weeks to get evaluated.

Having systemic symptoms, anaemia which is usually severe, low platelets, lymphadenopathy, and hepato-splenomegaly were the most frequent observations in most of our patients similar to many studies, but with different prevalence such as the CALLME1 study where the prevalence was for fever (75.5%), pallor (79.2%), lymphadenopathy (62.6%) (P = 0.0001 when compared to our study), hepatomegaly (59.5%), and splenomegaly (60.8%), and the Brazilian study where the prevalence was for anaemia (85%) with (35%) having severe anaemia of Hb < 7 g/dl (P > 0.05), low platelet counts of less than (100 × 10^9^ cells/L) in (65%) of the patients with (10.5%) having platelet counts less than (20 × 10^9^ cells/L) (P = 0.0177), lymphadenopathy (43.4%) (P = 0.0003 when comparing these numbers to our study), hepatomegaly (63%), and splenomegaly (57.8%). Our study had significantly higher prevalence of the aforementioned factors when comparing to these studies.

The most frequent haemoglobin level group in our study was (11–7 g/dL) and platelet count group was (50–150 × 10^9^ cells/L). These were within ranges of CALLME1 study where mean haemoglobin level was (7.9 g/dL) and platelet count mean was (66.1 × 10^9^ cells/L) and the Brazilian study where mean haemoglobin level was (8.24 g/dL). However, high WBC count was only found in half of our sample which is similar to what was found in the Brazilian study where the average WBC counts at diagnosis was (31.8 × 10^9^ cells/L).

ALL has many factors for negative prognosis such as high WBC count when presenting, CD10 negativity, lymphadenopathy and having extra-medullary disease^1^. Most patients in our study had either abnormal platelet counts or low haemoglobin level when diagnosed with only (2.0%) of the patients having normal levels for both which means that they can be used when patients presenting with ALL is speculated at crisis time such as the war in Syria to prioritise patients; Our findings are similar to what was found in Brazil where (4%) of the patients had normal FBC (P > 0.05 when comparing to this study). Positive findings on CXR were found in (18.4%) in our study which was higher than what was found in the Brazilian study (11.8%) (P > 0.05 when comparing to this study). Other prognostic factors include age, gender, and race^1^. Patients in the older age group (10–14) were found to have a worse prognostic risk (85.7% of them had high risk). However, most studies showed a good prognosis for the age group (1–9)^1^.

### Other variables

Overall, parents’ educational level was low in ALL patients as more than the half of fathers and mothers had low educational level. Positive family history in our study was lower than in the Tehran study (16.3%) (P > 0.05). In our study, high-risk patients had more positive family history, but more negative CD10 and higher prevalence of L2. Moreover, L2 was also correlated with more negative CD10, higher parents’ educational level and worse prognostic risk. This finding is similar to a study which found that high educational level of mothers was associated with higher risk of ALL^16^.

T-ALL and lymphadenopathies were more commonly found in males (82.5% of T-ALL cases were males). However, males required a shorter period before evaluation which could reflect that symptoms might have been more severe with males. T-ALL, high risk category and L2 were also found more frequently in the older patients, reflecting a poorer prognosis in these patients. High WBC count at diagnosis, high risk and more findings on CXR were found more in T-ALL patients than B-ALL, but lower haemoglobin in B-ALL patients was more frequent than T-ALL. L2 was also found more frequently in T-ALL patients.

T-ALL is known to affect males more than females which can explain that being a male was correlated with a higher risk^17^ although L1 and L2 affected both genders equally in our study (P > 0.05), and L2 was also correlated with a poorer prognosis. FAB classification in our patients showed a higher rates of L2 and L3 (P = 0.0001 when comparing our result with other studies) in comparison with the CALLME1 study where FAB classification was L1 = 77.4%, L2 = 20.4% and L3 = 21%, and with a Brazilian study L1 = 83% and L2 = 17%^15^, but L1 incidence in our study was close to what was found in Tehran L1 = 57.6% and L2 + L3 = 42.4% (P > 0.05). However, another study found that L1 accounted for (85–89%) of the cases, L2 (14.1%) and L3 (0.8%)^18^.

T-ALL prevalence in our study was higher than the CALLME1 study where T-ALL = 14.8% (P = 0.079 when comparing these two studies), and the Brazilian study where T-ALL = 10.5% (P = 0.0867 when comparing these two studies) which reflect multiple factors that could be affecting these findings. Higher risk patients were more frequent in Syria (48.4%) than what was found in the CALLME1 study where high risk patients comprised (36.0%) (P = 0.0108) and what was found in Brazil where high risk patients comprised (46%) (P > 0.05). Therefore, ALL patients in Syria have more frequently poor prognosis which could be due to other factors being involved in the period of the study such as the war. T-ALL is known for its poorer prognosis^12,19^. This all could explain the very high prevalence of high-risk ALL in Syria as these poor prognostic factors had a higher prevalence when comparing our study with other studies. It is crucial to study all prognostic factors to conduct an adequate treatment plan, so that patients are not under- or overtreated^20^. All the prognostic factors should be determined prior to treatment as an intense treatment protocol can eliminate the effect of some of the unfavourable factors and decrease relapse as protocols differ among risk groups^1,21,22^. T-ALL and L3 (Burkett) incidence can be related to virus exposure^23^. Using FAB system is convenient in developing countries as it is easy to conduct in regular labs and does not require much resources^10^, and it remains effective despite cytogenetic tests as it can add diagnostic accuracy in some cases^9^. L2 was found to have a higher relapse and mortality rate^18^ which is similar to our finding of L2 being correlated with higher risk. A weak or negative CD 10 expression is correlated with *ZNF384*, and *KMT2A* in blasts which often express high levels of *FLT3* rearrangement, *t(4;11)(q21;q23)* in particular which accompanies a poorer outcome^1^. However, leukemic cells which demonstrate a germline of *KMT2A* gene configuration are correlated with positive CD10 expression in precursor-B ALL and have a better outcome^1^. Nevertheless, CD10 prognostic significance independently from *KMT2A* rearrangement is not clear^1^.

The much higher L2 and high risk prevalence comparing to other studies may reflect an underlying cause, such as from war or environment as many practices in Syria may contain leukemogenesis such as unprotected pesticide usage, mate drinking and hookah smoking, mainly in low educational level population^24^. It is suggested that the protocols that were developed in the advanced centres might increase the rate of death as these protocols are not adjusted to the local conditions of low- and mid-income countries^2,3^, and therefore more studies are required in developing countries such as Syria for adjustment of protocols that change ALL variables. Although the cost of treatment in Syria is covered, there is data suggesting that families within low SES are correlated with worse prognosis in children as determination of indirect costs is difficult^8^ which can explain having lower educational parents was correlated with poorer prognosis in our study.

In conclusion, in this study we have discussed multiple features and risk factors of ALL and compared characteristics of ALL children in Syria in the Middle East with multiple studies from the Middle East and multiple regions across the globe. The data covered most aspects of ALL and its prevalence in addition to factors which are correlated with worse prognosis such as L2 FAB classification, negative CD10, male gender, T-ALL, and low parental educational level. The results suggest high T-ALL, L2, and high risk prevalence which could reflect underlying factors and poor survival rates, especially that treatment protocols may have a higher mortality in developing countries when not adjusted to local variables. The results also suggests that having normal haemoglobin and platelet count can be used for quick screening in crisis time like in Syria for prioritising patients.

This is the first detailed study to demonstrate the epidemiology of ALL in Syria and its relation to other factors. It also suggests common risk factors that might worsen the prognosis while comparing with multiple studies from different countries. This study was also conducted at war-torn Syria which also could be the factor for this phenomenon. It also enforces the significance of FAB classification and its relation to higher risks of ALL. The different findings also enforce the importance of local studies in developping countries as they might have considerably different factors than the developed countries.

## Data Availability

Data will be available on request from the corresponding author.
